# Constructing high-order functional connectivity network based on central moment features for diagnosis of autism spectrum disorder

**DOI:** 10.7717/peerj.11692

**Published:** 2021-07-06

**Authors:** Qingsong Xie, Xiangfei Zhang, Islem Rekik, Xiaobo Chen, Ning Mao, Dinggang Shen, Feng Zhao

**Affiliations:** 1 School of Computer Science and Technology, Shandong Technology and Business University, Yantai, Shandong, China; 2 School of Science and Engineering, Computing, University of Dundee, Dundee, Dundee, United Kingdom; 3 BASIRA Lab, Faculty of Computer and Informatics, Istanbul Technical University, Istanbul, Istanbul, Turkey; 4 Department of Radiology, Yantai Yuhuangding Hospital, Yantai, Shandong, China; 5 School of Biomedical Engineering, ShanghaiTech University, Shanghai, China; 6 Shanghai United Imaging Intelligence Co., Ltd., Shanghai, China; 7 Department of Artificial Intelligence, Korea University, Seoul, South Korea

**Keywords:** Autism spectrum disorder, Functional magnetic resonance imaging, Functional connectivity, High functional connectivity network, Low functional connectivity network, Dynamic functional connectivity network, Central moment feature, Feature extraction, Feature selection, Cross validation

## Abstract

The sliding-window-based dynamic functional connectivity network (D-FCN) has been becoming an increasingly useful tool for understanding the changes of brain connectivity patterns and the association of neurological diseases with these dynamic variations. However, conventional D-FCN is essentially low-order network, which only reflects the pairwise interaction pattern between brain regions and thus overlooking the high-order interactions among multiple brain regions. In addition, D-FCN is innate with temporal sensitivity issue, i.e., D-FCN is sensitive to the chronological order of its subnetworks. To deal with the above issues, we propose a novel high-order functional connectivity network framework based on the central moment feature of D-FCN. Specifically, we firstly adopt a central moment approach to extract multiple central moment feature matrices from D-FCN. Furthermore, we regard the matrices as the profiles to build multiple high-order functional connectivity networks which further capture the higher level and more complex interaction relationships among multiple brain regions. Finally, we use the voting strategy to combine the high-order networks with D-FCN for autism spectrum disorder diagnosis. Experimental results show that the combination of multiple functional connectivity networks achieves accuracy of 88.06%, and the best single network achieves accuracy of 79.5%.

## Introduction

Autism Spectrum Disorder (ASD) is a childhood nervous system developmental disorder and persists into adulthood. Its main clinical manifestations include social and communication difficulties, restricted interest, repetitive behavior, and language developmental disorder. According to the latest report by the Centers for Disease Control and Prevention, about one in 59 American children is affected by some forms of ASD and four times more common among boys than among girls. To date, there is no effective way to completely cure ASD, individuals with ASD suffer from lifelong illness (Elisabeth Fernell & Gillberg, 2013; Ecker, Bookheimer & Murphy, 2015). Therefore, it is of great significance to diagnose and intervene ASD as early as possible for the improvement of patients’ quality of life. Accurate brain imaging-based ASD diagnosis is still challenging since brain anatomical and functional changes in this stage are considerably subtle. By far, previous studies (Wang et al., 2020; Huang et al., 2018) have already indicated that resting-state functional magnetic resonance imaging (RS-fMRI) can serve as a promising imaging technique for ASD diagnosis.

RS-fMRI is an emerging neuroimaging technology, which uses blood oxygenation level-dependent (BOLD) signals to explore the biomarkers of nervous system diseases and has been successfully applied to the diagnosis of ASD. Functional connectivity (FC), defined as the temporal correlation of BOLD signals in different brain regions, can exhibit how structurally segregated and functionally specialized brain regions interact with each other (Friston et al., 1993; Michael, 2008). FC network has been of great importance for discovering the functional organization of human brain and searching for the biomarkers of the neuropsychiatric disorders, such as Alzheimer’s disease (Chen et al., 2017; Hao et al., 2017) and autism spectrum disorder (ASD) (Zhao et al., 2018; Wee, Yap & Shen, 2016). Currently, researchers have proposed various FC network modeling methods for ASD assisted diagnosis (Liu & Huang, 2020; Zhao et al., 2020; Zhao et al., 2021). For example, Liu & Huang (2020) estimated the severity of ASD by multivariate model analysis, and they found that some FCs suffer from abnormal alterations in ASD patients. Zhao et al. (2021) proposed a unit-based personalized fingerprint feature selection (UPFFS) strategy and applied to ASD, they found that the top selected discriminative brain regions by UPFFS are related to visual processing, social cognition, and emotional expression which is associated with ASD. Overall, previous studies have shown that FC networks have great potential for revealing FC deficits and finding abnormal brain regions in ASD patients.

Due to the complexity of human brain, the FC relationship among different brain regions may be reflected at multiple levels. However, many previous studies usually used the single characteristic of FC to construct FC network (Zhang et al., 2016; Wee et al., 2016; Zhang et al., 2018). For instance, Wee et al. (2016) proposed a classification method based on a sparse temporal dynamic network, and suggested that the temporal dynamic information is crucial for accurate diagnosis of neurological disorders, but it failed to capture the complex high-order FC pattern. Zhang et al. (2016) constructed a high-order FC network to capture this second-level relationship using inter-regional resemblance of the FC topographical profiles, it is more sensitive to group difference, able to better capture individual variability, and able to show more prominent modular structures. However, this method is based on the assumption and FC is static pattern of it, which ignores the dynamic characteristic of FC. The above two types of FC network are analyzed from the view of dynamic and static high-order, respectively. Therefore, how to effectively simulate the complex FC interaction pattern which combines dynamic and high-order is still an important challenge.

Sliding window method is the popular method to construct dynamic FC network (D-FCN). However, D-FCN reflects the pair-wise dynamic FC relationship between brain regions and ignores the FC interaction pattern among multiple brain regions, in such a sense, the D-FCN is called low-order D-FCN (LoD-FCN). Correspondingly, the FC network which can reflect the FC relationship among multiple brain regions is called high-order FC network. LoD-FCN is sensitive to the chronological order of its subnetworks, which makes it difficult to make consistent and meaningful comparisons among different subjects (Chen et al., 2016). Specifically, the FC subnetwork of LoD-FCN depends on its relative position in the whole time series, if the relative positions of the two FC subnetworks are switched, the LoD-FCN will be changed. This leads to the sensitivity of LoD-FCN to its subnetwork, which limits its use in comparative studies. In order to eliminate the sensitivity, the central moment method was used to extract features from LoD-FCN (Zhao et al., 2020, 2021). The central moment feature is a common translation invariant feature, which reflects the shape information of the target and is often used in feature extraction of sequences or waveforms. In theory, the change characteristics of a random sequence can be better represented by central-moment features, the second-order central moment (i.e., variance) can reflect the fluctuation level, third-order central moment can reflect the skewness, and the fourth order central moment can reflect the kurtosis, and so on. Note that the first central moment is equivalent to 0 in the mathematical sense, we use the mean instead of the first central moment in this study.

Inspired from the LoD-FCN and the central moment method, we propose a novel high-order FC network framework which reflects the interaction of low-order dynamic FCs on the moment-level to measure brain high-order FC pattern. Specifically, we firstly construct a LoD-FCN by the sliding window strategy, and then, the central moment method is employed to extract central moment feature FC network (CM-FCN) from LoD-FCN. We regard the row of the CM-FCN as the FC topographical profile of a special brain region, reflecting the central-moment features of the FC time series from the LoD-FCN. Then, the high-order FC is computed between two FC profiles. Each order central moment reflects the statistical information of FC dynamic changes, and multiple high-order FC networks can be constructed by changing the order number.

Our motivation is based on the hypothesis that FC of ASD children may change at the moment-level, which may be due to miswiring during abnormal development. CM-FCN reflects the topographical information of the center moment feature of dynamic FC, which provides rich discriminative information for disease recognition and classification (Zhao et al., 2020). However, CM-FCN is essentially a low-order network, since it captures pair-wise FC topographical information. The current propose mothed provides diagnostic information for ASD by calculating the correlation between the central moment statistical features of brain FC dynamic changes. This method includes both dynamic characteristic of low-order FC and complex high-order FC pattern.

**Figure 1:**
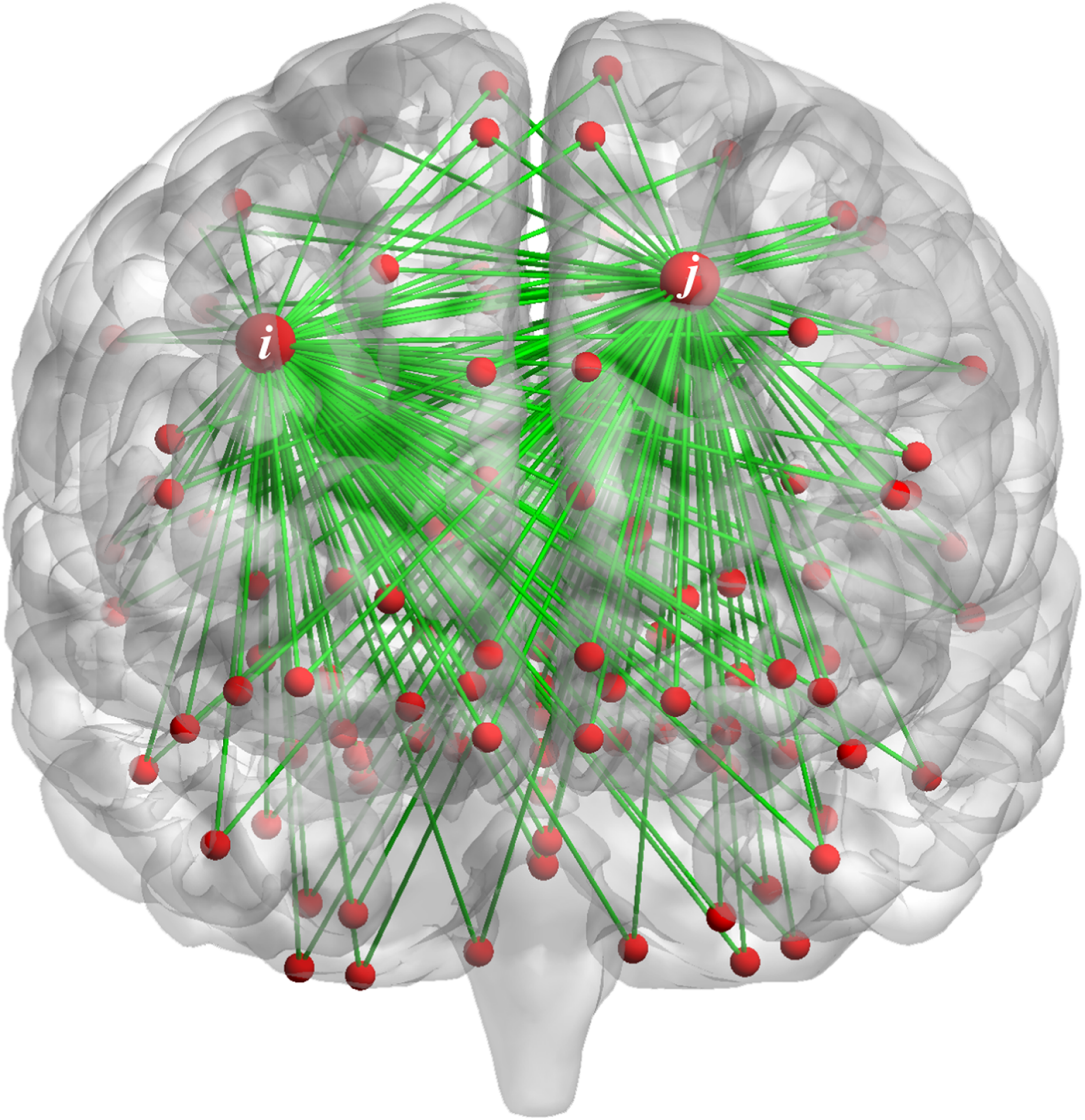
The FC of the *i*-th (*j*-th) brain region and other brain regions.

Taking variance as an example, the variance reflects the fluctuation level, the larger the variance value of FC changes along time, the more unstable FC in the during scan. As show in Fig. 1, the FC between the *i*-th (*j*-th) brain region and other brain regions is dynamic. Whether the stability of FC between the *i*-th brain region and other brain regions is related to the FC of *j*-th brain region with other regions, which can be reflected in the correlation strength of variances. We propose that the FC changes between the *i*-th brain region and other brain regions may related to the FC changes between the *j*-th brain region and other brain regions. This interaction by calculating the correlation of central moment features may provide important information for the diagnosis of ASD.

In summary, our high-order FC network has the following advantages: (1) it takes the FC time-varying characteristics into account since it takes the LoD-FCN as the infrastructure; (2) multiple high-order FC networks can be constructed by changing the order of the central moment to represent the interaction patterns of brain regions; (3) the discriminability can be further improved by integrating multiple high-order FC networks.

## Materials & methods

In this paper, lowercase letters (e.g., *x*) denote scalars, lowercase bold letters (e.g., ***x***) denote vectors, and uppercase bold letters (e.g., ***D***) denotes matrices or FC networks. All FC networks are stored in matrices, where each column (or row) denotes a vertex of the corresponding FC network and the element denotes the associated weight of an edge between two vertices.

**Figure 2:**
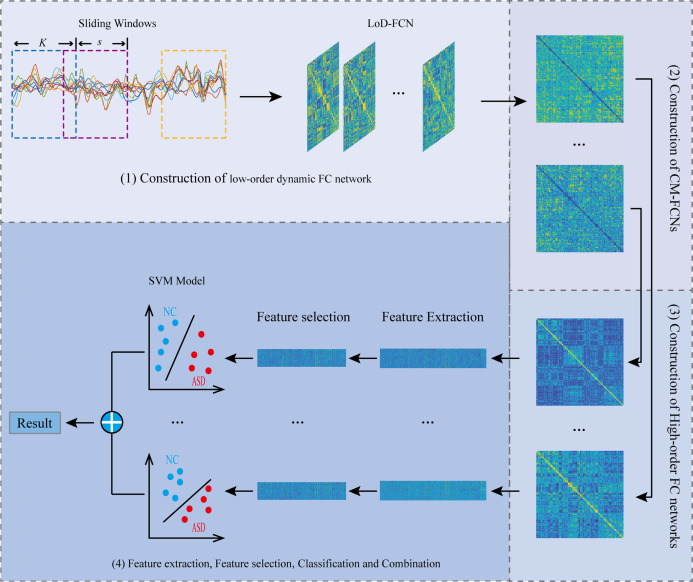
The flowchart of the proposed framework.

The flowchart of the proposed framework is illustrated in Fig. 2. Overall, there are four steps, including (1) Constructing LoD-FCN; (2) Constructing CM-FCNs; (3) Constructing high-order FC networks; (4) Feature extraction, feature selection, classification, and combination.

### Data acquisition and preprocessing

In this study, we conducted experiments on the Autism Brain Imaging Data Exchange (ABIDE) database (Di Martino et al., 2014). We chose 45 ASD patients (36 males and 9 females) and 47 NC subjects (36 males and 11 females) aged between 7 and 15 years old, scanned at New York University (NYU) Langone Medical Center. These subjects were sociodemographic-matched, where there were no significant differences (*p* > 0.05) in gender, age, and full intelligence quotient (FIQ) between ASD group and NC group. The demographic information is summarized in Table 1. The diagnosis of ASD subjects was based on the autism criteria sets in Diagnostic and Statistical Manual of Mental Disorders, 4th Edition, Text Revision (DSM-IV-TR). In this work, only RS-fMRI data were utilized for diagnostic study.

**Table 1:** Demographic information of the subjects.

	ASD	NC	*p*-values
Gender (M/F)	36/9	36/11	0.2135^a^
Age (mean ± SD)	11.1 ± 2.3	11.0 ± 2.3	0.773^b^
FIQ (mean ± SD)	106.8 ± 17.4	113.3 ± 14.1	0.0510^b^
ADI-R (mean ± SD)	32.2 ± 14.3^c^	–	–
ADOS (mean ± SD)	13.7 ± 5.0	–	–

**Notes:**

ASD, Autism Spectrum Disorders; NC, normal control; M, male; F, female; FIQ, Full Intelligence Quotient; ADI-R, Autism Diagnostic Interview-Revised; ADO, Autism Diagnostic Observation Schedule.

a The *p*-value was obtained by χ^2^-test.

b The *p*-value was obtained by two-sample two-tailed *t*-test.

c Two patients do not have the ADI-R score.

All subjects we selected underwent a 6-min scan using a 3T Siemens Allegra scanner at NYU Langone Medical Center. During the RS-fMRI scans, all subjects were asked to relax with their eyes open, and gaze at a white fixed cross in the middle of a black background projected onto a screen to focus their attention prevent meditation with eyes closed, which ensured the subjects do not have violent neural activity. During RS-MRI scanning, eye movement was monitored by an eye tracker. When acquiring images, the following parameters were used: TR/TE = 2,000/15 ms, flip angle = 90°, 33 slices per volume, 180 volumes per scan, voxel thickness of 4.0 mm. The mean frame-wise displacement (FD) was computed to describe head motion for each individual. Individuals with mean FD larger than 1 mm were excluded for reducing the negative effect of head motion (Lin et al., 2015; Ray, Gohel & Biswal, 2015).

Statistical Parametric Mapping (SPM8) software was adopted to preprocess the acquired RS-fMRI data (http://www.fil.ion.ucl.ac.uk/spm/software/spm8/). The first 10 RS-fMRI images of each subject were discarded for magnetization equilibrium. Then, the remaining images were spatially normalized into the Montreal Neurological Institute (MNI) template space with resolution of 3 × 3 × 3 mm^3^. Other corrections were further conducted, including the regression of nuisance signals (ventricle, white matter, global signals, and head motion with Friston 24-parameter model), signal detrending, and band-pass filtering (0.01–0.08 Hz) (Satterthwaite et al., 2013; Yan et al., 2013; Cordes et al., 2001; Sophie et al., 2008; Tomasi & Volkow, 2010). Each brain image was then parcellated into 116 regions according to the Automated Anatomical Labeling (AAL) atlas (Tzourio-Mazoyer et al., 2002). Finally, the average of RS-fMRI time series within each brain region was calculated, which was treated as the data matrix }{}{\bf X} \in {R^{170 \times 116}} for subsequent processing, where 170 denotes the total number of temporal image volumes and 116 denotes the total number of brain regions.

### Construction of low-order dynamic FC network

Let }{}{\bf{x}_i} = {\left( {{x_{i1}},{x_{i2}}, \cdots ,{x_{iM}}} \right)^T}\left( {i = 1,2, \cdots ,R} \right) denote the RS-fMRI time series associated with the *i*-th brain region, where *M* = 170 is the number of image volumes after discarding the first 10 volumes, and *R* is the total number of brain regions.

**Figure 3:**
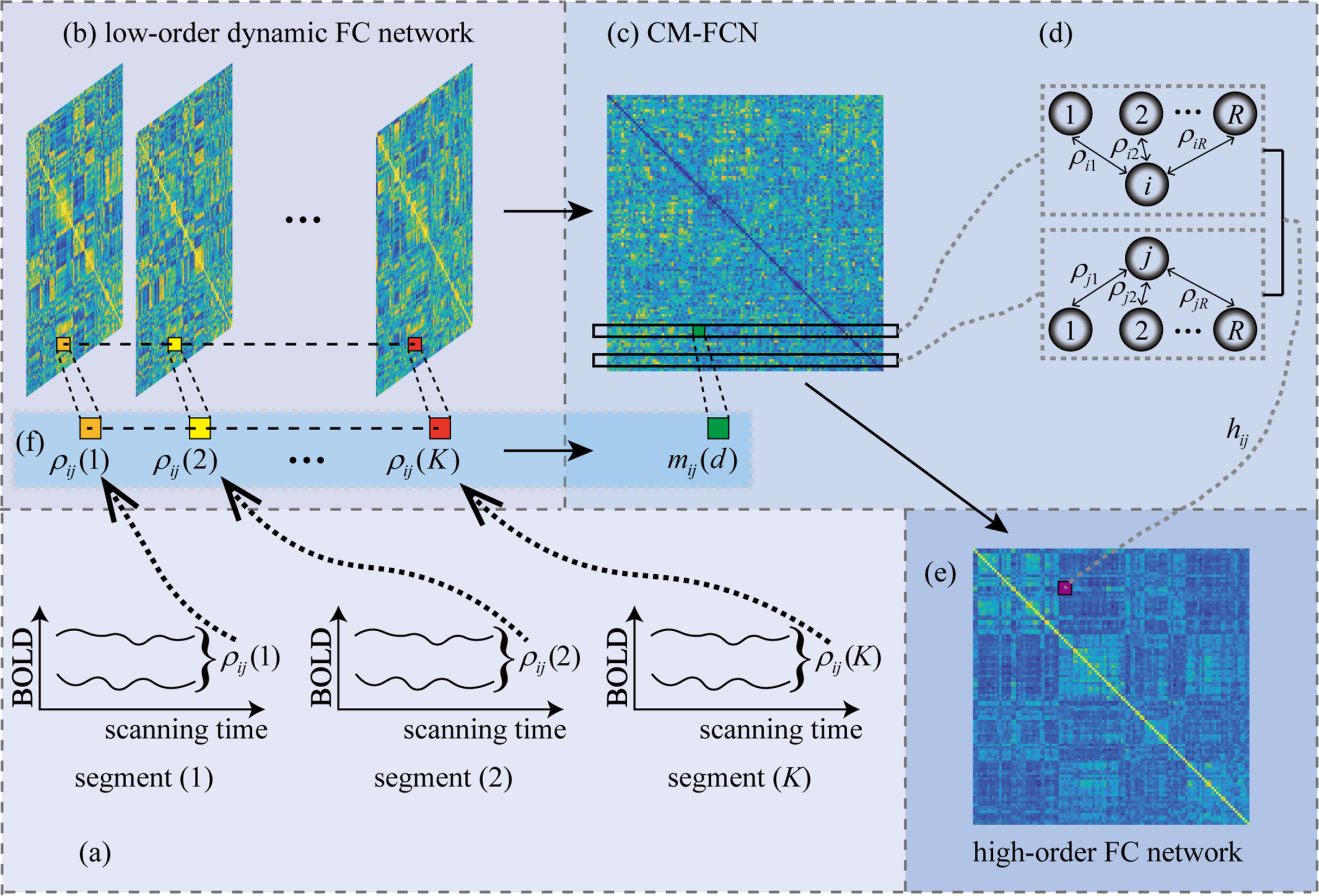
The flowchart of LoD-FCN and high-order FC network construction. The ***ρ**_ij_*(*k*) denotes the short-time correlation between *i*-th and *j*-th ROIs in *k*-th window, 1 ≤ *k*≤ *K*; The *m_ij_*(*d*) is the *d*-th order central moment feature of the FC time series, i.e., ***ρ**_ij_* = (*ρ_ij_*(1), *ρ_ij_*(2), …, *ρ_ij_*(*K*)); The *h_ij_* denotes high-order FC that obtained by calculating the correlation between the *i*-th and *j*-th rows of CM-FCN.

We employ the sliding window strategy to generate LoD-FCN for encoding the nonstationary interactions between different brain regions. Fig. 3 illustrates the steps of LoD-FCN construction. Specifically, a sliding window with fixed length is utilized to partition the RS-fMRI time series into }{}K = \left( {M - W} \right)/s + 1 overlapping segments (Fig. 3A), where *W* is the length of sliding window and *s* is the translational step size of sliding window. Let }{}{\bf{x}_i}\left( k \right) (}{}1 \le k \le K) denote the sub-series of the *i*-th brain region in the *k*-th window. Then, for each segment, the short-time correlation between the *i*-th and the *j*-th brain region is computed as:

(1) }{}{\rho _{ij}}\left( k \right) = corr\left( {{\bf{x}_i}\left( k \right),{\bf{x}_j}\left( k \right)} \right)

Thus, the subnetwork can be constructed as }{}{{\bi D}_{\rm Lo}}\left( k \right) = {\left[ {{\rho _{ij}}\left( k \right)} \right]_{1 \le i,j \le R}} (}{}1 \le k \le K), the LoD-FCN can be denoted as }{}{\bi D}_{\rm Lo}} = \left[ {{{\bi D}_{\rm Lo}}\left( 1 \right), \cdots ,{{\bi D}_{\rm Lo}}\left( k \right), \cdots ,{{\bi D}_{\rm Lo}}\left( K \right)} \right] (Fig. 3B). For two specific brain regions, }{}{{\bf {\it \rho} }_{ij}} = \left( {{\rho _{ij}}\left( 1 \right), \cdots ,\; {\rho _{ij}}\left( k \right), \cdots ,{\rho _{ij}}\left( K \right)} \right) can reflect the dynamic correlation between the *i*-th and the *j*-th brain regions along time (see Fig. 3F).

Note that the infrastructure of LoD-FCN will be destroyed if we change the relative position of its subnetwork. The reason is that RS-fMRI scans along time, if the relative position of two subnetworks is switched, the chronological structure of RS-fMRI will be changed. Therefore, the subnetworks must be arranged in strict time order, i.e., LoD-FCN is sensitive to the chronological order of its subnetworks.

### Construction of CM-FCN

As mentioned above, LoD-FCN is sensitive to the chronological order of its subnetworks. In order to eliminate the sensitivity, we adopt the central moment method to extract the central moment feature FC network (CM-FCN) from LoD-FCN (see Fig. 3C). The *d*-th central moment can be calculated by Eq. (2).

(2) }{}{m_{ij}}\left( d \right) = \root d \of {\displaystyle{{\mathop \sum \nolimits_{k = 1}^K {{\left( {{\rho _{ij}}\left( k \right) - {{{\bar {\bf \rho} }}_{ij}}} \right)}^d}} \over K}}

where }{}{{\bar {\bf\rho} }_{ij}} = \displaystyle{{\mathop \sum \nolimits_{k = 1}^K {\rho _{ij}}\left( k \right)} \over K} is the average of all elements in }{}{\bf{\rho }_{ij}}. Thus, we can get the *d*-th order CM-FCN as }{}{\bf CM}\left( d \right) = {\left[ {{m_{ij}}\left( d \right)} \right]_{1 \le i,j \le R}}, and construct multiple CM-FCNs by varying *d*. Specially, since }{}{m_{ij}}\left( d \right) is equal to 0 when *d* = 1, we use the mean value (i.e., }{}{{\bar {\bf\rho} }_{ij}}) instead of the first order central moment.

In }{}{\bi CM}\left( d \right), an element, i.e., }{}{m_{ij}}\left( d \right), denotes the fluctuation characteristic of FC along the scanning time in a pair of brain regions, and the *i*-th row vector (denotes as }{}{\bi{m}_i}\left( d \right)) represents the characteristics of low-order dynamic FC for the *i*-th brain region and other brain regions (see Fig. 3D).

### Construction of high-order FC network

In order to find that how the fluctuation characteristics of FC interact with each other, we use the high-order FC, obtained by calculating the correlation between any two rows of }{}{\bf CM}\left( d \right), reflecting the high-order interaction. The high-order FC }{}{h_{ij}}\left( d \right) is calculated by:

(3) }{}{h_{ij}}\left( d \right) = corr\left( {{\bi{m}_i}\left( d \right),{\bi{m}_j}\left( d \right)} \right)

where the }{}{\bi{m}_i}\left( d \right) = \left( {{m_{i1}}\left( d \right),{m_{i2}}\left( d \right), \ldots ,{m_{iR}}\left( d \right)} \right) denotes the row of *d*-th order CM-FCN, it means that the central moment features of dynamic FC of *i*-th brain region with other brain regions.

Thus, we can get a high-order FC network as }{}{\bi Ho}\left( d \right) = {\left[ {{h_{ij}}\left( d \right)} \right]_{1 \le i,j \le R}}, when the value of *d* is different, the high-order interaction information of high-order FC network reaction is also different. For instance, the second-order central moment (i.e., variance) feature can reflect the fluctuation level, the larger the variance value of FC time series, the more unstable FC in during scanning. Thus, the }{}{h_{ij}}\left( 2 \right) reflects that whether the stability of FC between the *i*-th brain region and other brain regions relates to the FC stability of the *j*-th brain region and other brain regions.

### Feature extraction, feature selection, classification

For the *l*-th subject, we use its corresponding high-order FC matrices }{}{\bi Ho}\left( d \right) as raw features. Considering the symmetry of each FC matrix, we only vectorize its lower off-diagonal triangular part to define the feature vectors, i.e., }{}{\bi y}^l\left( d \right), for representing the *l*-th subject. The dimensionality of }{}{\bi{y}^l}\left( d \right)\; \left( {1 \le d \le 10} \right) is }{}{{M\left( {M - 1} \right)} \over M}, where *M* denotes the number of brain regions.

The feature vectors }{}{\bi{y}^l}\left( d \right) extracted from high-order FC network may include irrelevant or redundant features for ASD diagnosis. Therefore, it is necessary to further select the most-relevant features. To reduce noise features, we perform *t*-test and }{}{L_1}-norm regularized least squares regression, known as LASSO (Tibshirani, 1996), for feature selection. Specifically, for each feature from }{}{\bi{y}^l}\left( d \right), we perform a two-sample *t*-test between NC and ASD subjects, due to its simplicity and efficiency. Then, we select the features only with their *p*-values smaller than a certain threshold, denote as }{}{{\hat {\bi{y}} (d). After *t*-test, we adopt the LASSO to further optimize the feature subset. Let }{}{\bf{\omega }_i} = {\left( {{w_{i1}},{w_{i2}}, \cdots ,{w_{ic}}} \right)^T} represent the weight vector for the feature selection task and }{}{I^{\left( l \right)}} is the class labels of }{}{{\hat {\bi{y}} \left( d \right), where }{}{I^{\left( l \right)}} = 1 when the *l*-th subject is ASD and }{}{I^{\left( l \right)}} = - 1 when the *l*-th subject is NC. Mathematically, the LASSO model can be described as follows:

(4) \displaystyle{1 \over 2}\mathop \sum \limits_{l = 1}^L\left\| {I^l} - \left\langle {{\hat {\bi{y}},{\bf{\omega }_i}}} \right\rangle\right\| _2^2 + {\rm \lambda }\|{\bf{\omega }_i}\|_1

where 〈·, ·〉 denotes the inner product operator, and λ is a regularization term. A value of λ can make the solution }{}{\bf{\omega }_i} sparser. By setting a proper value for λ, we can achieve sparse feature selection, where features corresponding to the non-zero elements of }{}{\bf{\omega }_i} are retained. For simplicity, let }{}{\bi{ y}^{\hskip -.4pc\cdots l}\left( d \right) represent the final feature set selected from the feature vector }{}{\bi{ y}^{\hskip -.4pc\cdots l}\left( d \right).

After selecting the most-relevant features with *t*-test and LASSO, we use Support Vector Machine (SVM) (Cortes & Vapnik, 1995) with simple linear kernel for disease identification. SVM seeks a maximum margin hyperplane to separates the samples of one class from the another, meanwhile minimizing the classification errors. The empirical risk on the training data and the complexity of the model can be balanced by a hyperparameter, thus ensuring the good generalization ability of the unknown data. Herein, we construct a SVM model for each high-order FC network.

### Experimental settings

In this work, we used the 10-times five-fold cross-validation strategy to evaluate the effectiveness of the proposed method. All data were divided into five subsets of the same size, with one part of each subset as the test set and the other four parts as the training set. To avoid any possible bias in fold selection, the entire five-fold cross-validation process was repeated 10 times, with a different random partitioning of samples each time. Note that the hyperparameters in the process of the “Feature extraction, Feature selection, Classification” were tuned based on the training subjects by a nested five-fold cross-validation in order to avoid the effect of overfitting. Finally, the average statistics of the 10 repetitions were reported. To compare different methods, we used the following performance indexes: accuracy (ACC), sensitivity or true positive rate (TPR), specificity or true negative rate (TNR), F1-score:

(5) }{}{\rm ACC} = \displaystyle{{TP + TN} \over {TP + FP + TN + FN}}\;

(6) }{}{\rm TPR} = \displaystyle{{TP} \over {TP + FN}}\;

(7) }{}{\rm TNR} = \displaystyle{{TN} \over {FP + TN}}

(8) }{}{\rm F}1 = \displaystyle{{2 \times TP} \over {2 \times TP + FN + FP}}

where *TP*, *TN*, *FP*, and *FN* indicate true positive, true negative, false positive and false negative, respectively.

Since we construct the LoD-FCN by sliding windows and construct Ho-FCN based on LoD-FCN, the window length (*W*) and translational step size (*s*) of the sliding window may have an impact on the performance of the high-order FC network. We set the range of *W* and *s* as }{}W \in \left[ {30,40, \cdots ,100} \right], }{}s \in \left[ {2,4, \cdots ,16} \right]. And we set the order of central moment from 1 to 10, i.e., }{}d \in \left[ {1,2, \cdots ,10} \right] for constructing multiple high-order FC networks.

## Results

### The performance on high-order FC network

The number of the windows is }{}K = \left( {M - W} \right)/s + 1, it can be seen that changing the sliding window length (*W*) and translational step size (*s*) alter the number of sliding windows. At the same time, the number of LoD-FCN’s subnetwork will also be different. Therefore, *W* and *s* affect the infrastructure of LoD-FCN, and then affect the structure of CM-FCNs. This may lead to changes in high-order FC network performance. It is a dilemma to choose an appropriate window length and step size, since the window length should be short enough to capture short-term fluctuations while long enough to allow robust FC estimation (Sakoglu et al., 2010). Thus, we optimize the performance of each network by adjusting the values of parameters *W* and *s*.

**Figure 4:**
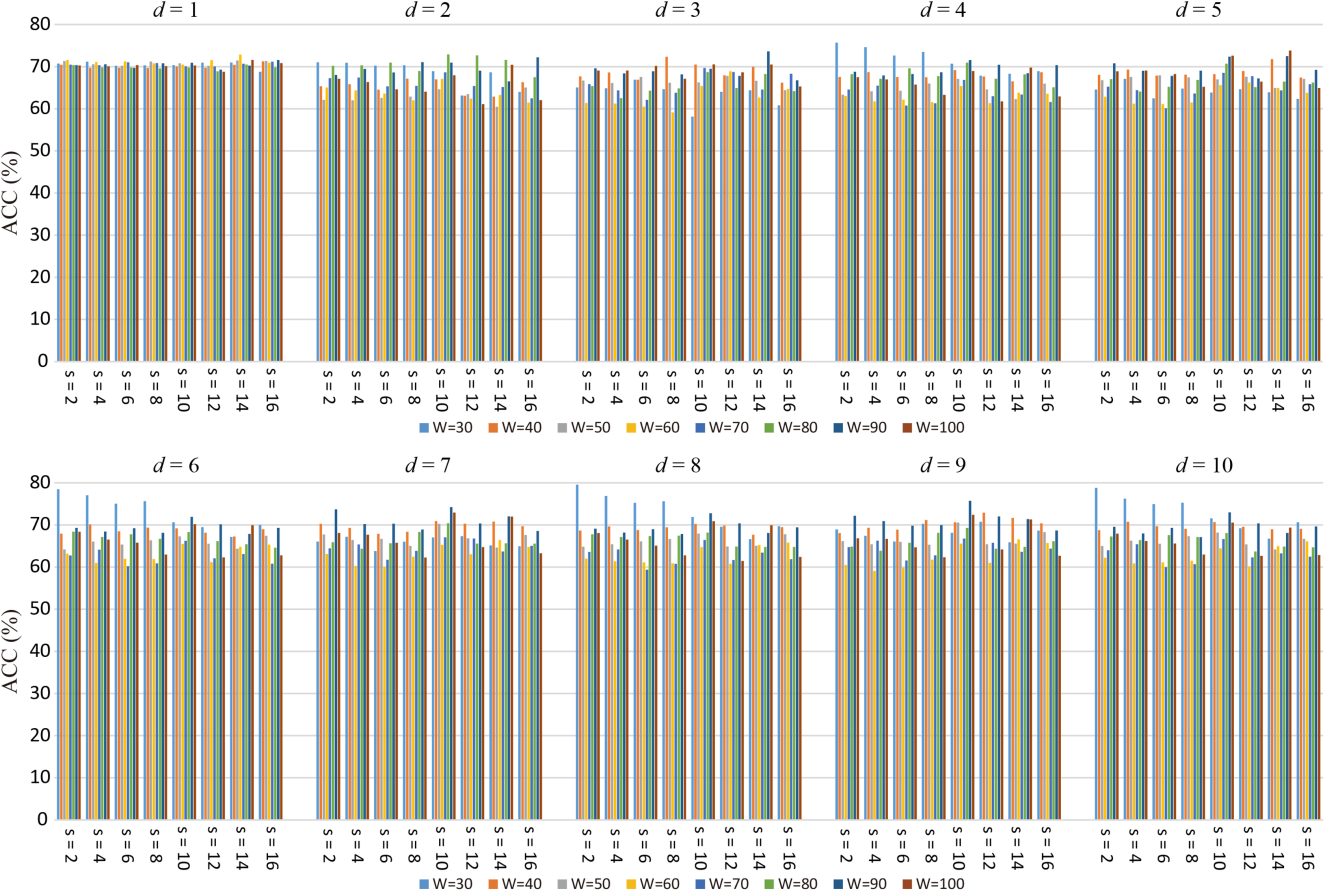
The average ACC of high-order FC networks using different combinations of *W* and *s*.

Figure 4 displays the ACC achieved by high-order FC networks using different combinations of *W*, *s*, and *d* values. We can see that when *W* = 30, *s* = 2 and *d* = 8, the highest classification accuracy is obtained and the ACCs are greatly influenced by *W*, *s*, i.e., classification performance is rather sensitive to these parameters. Each high-order FC network with varying *d* has different performance, indicating the high-order FC networks contains different level information for ASD diagnosis. Therefore, we can draw that it is necessary to select *W* and *s* carefully toward better understanding of dynamics in brains.

The best ACCs and corresponding *W*, *s* of the high-order FC networks are shown in Table 2, where ***Ho*** (*d*) \left( {1 \le d \le 10} \right) denotes the high-order FC network based on *d*-th order CM-FCN. We can see that there are some differences among the best ACC of high-order FC networks, the best result is achieved when the *d* = 8, which is about 6% higher than ***Ho*** (1). This indicates that the high-order FC networks contain different degrees information for ASD diagnosis. In addition, we notice that when the high-order FC networks achieve the best performances, the values of *W* (or *s*) are different, which indicates that the structure of LoD-FCN will affect the performance of the high-order FC network.

**Table 2:** The best performances of high-order FC networks.

Features	*W*	*s*	ACC (%)	TPR (%)	TNR (%)	F1 (%)
*Ho* (1)	60	14	72.86	72.00	73.68	72.30
*Ho* (2)	80	10	72.88	72.22	73.48	72.09
*Ho* (3)	90	14	73.63	72.88	74.33	72.69
*Ho* (4)	30	2	75.68	73.55	77.86	74.47
*Ho* (5)	100	14	73.83	79.55	68.48	74.64
*Ho* (6)	30	2	78.47	76.22	80.68	76.92
*Ho* (7)	90	10	74.19	73.77	74.60	73.51
*Ho* (8)	30	2	79.50	78.44	80.55	78.50
*Ho* (9)	90	10	75.67	74.00	77.28	74.78
*Ho* (10)	30	2	78.74	77.11	80.31	77.48

### The most discriminative features for ASD diagnosis

We used *t*-test, followed by LASSO regression, to identify the most discriminative features in high-order FC networks. In this study, we used the frequency, at which features are selected in all cross-validation cases, to quantify feature relevance to the target classification. The high-order FCs with the highest frequencies during the 10-times five-fold cross-validation were selected as the most discriminative connections. The reported results were based on the original AAL atlas (with 116 brain regions) (Tzourio-Mazoyer et al., 2002) for illustration.

**Figure 5:**
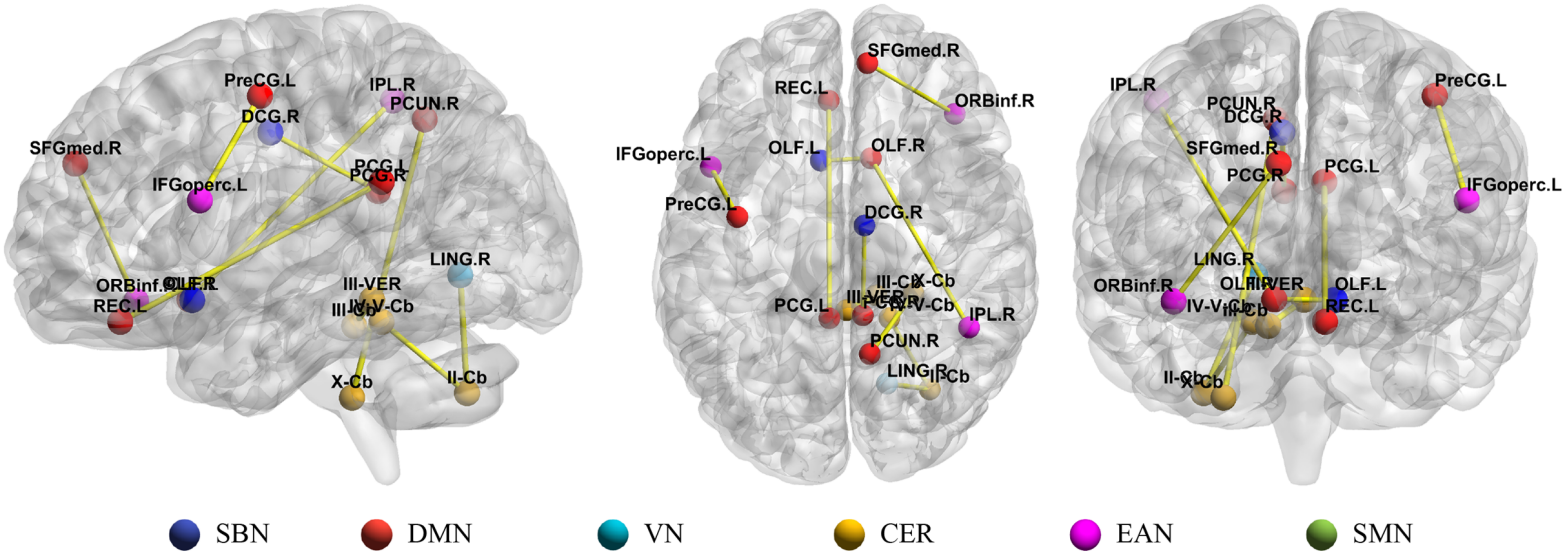
Illustration of top 10 most discriminative connections selected from *Ho* (8) with the highest frequencies. SBN, subcortical nuclei regions; DMN, default mode network; VN, visual network; CER, cerebellum; EAN, executive and attention network; SMN, sensorimotor network.

Since not all of the 10 networks we have constructed have good classification accuracy, in this subsection, we only analyze the high-order FC networks with the best classification accuracy (i.e., ***Ho*** (8)). In Fig. 5 and Table 3, we show the results the top 10 most discriminative features from ***Ho*** (8), where each link corresponds to a connectional feature. Since the proposed high-order FC represents the correlation between the central moment characteristics of the *i*-th brain region with other brain regions and the *j*-th brain region with other brain regions (as shown in Fig. 3D), for simplicity, we only visualize the connection between the *i*-th and the *j*-th brain region. Moreover, we calculate the frequency of the 10 most discriminative features which selected from ***Ho*** (8). The frequency is defined as the ratio of occurrence for brain region pairs in 10-times five-fold cross-validations. For example, if a feature is selected 49 times, then its frequency is 49/50 = 0.98, the related detailed information is shown in Table 3, where “.L” and “.R” denote the brain region belong to left and right hemisphere, respectively. To evaluate the significant differences between ASD and NC, the *p*-value at the 5% significance level of each discriminative brain region pair computed based on two sample *t*-test is also listed in Table 3. The *p*-values of discriminative features identified by our method are smaller than 0.01, showing the significant between-group difference individually.

**Table 3:** The 10 most discriminative features from *Ho* (8).

Features	Frequency	*p*-value
III-Cb & III-VER	1.00	0.000
PreCG.L & IFGoperc.L	0.98	0.001
X-Cb & PCUN.R	0.96	0.000
II-Cb & LING.R	0.94	0.001
REC.L & PCG.L	0.94	0.000
OLF.R & IPL.R	0.88	0.000
II-Cb & IV-V-Cb	0.86	0.003
ORBinf.R & SFGmed.R	0.82	0.004
DCG.R & PCG.R	0.84	0.002
OLF.L & OLF.R	0.82	0.007

In the previous study, 116 brain regions in AAL atlas were divided into six common functional networks according to BrainNet Viewer software (Xia et al., 2013): the default mode network (DMN), the execution and attention network (EAN), the sensorimotor network (SMN), the visual network (VN), the subcortical nuclei (SBN) regions and the cerebellum (CER). As shown in Fig. 5, half of the discriminative brain regions selected by our method came from DMN and CER.

## Discussion

### The influence of sliding window on high-order FC network

Due to the high spatial resolution, fMRI has become a powerful tool for studying human brain. However, the temporal resolution of fMRI is limited by the hemodynamic response function (HRF), which is usually sampled every few seconds. Sliding window method is a commonly used approach to obtain dynamic FC based on RS-fMRI, which is based on a temporal locality assumption. For a time series, if the rate of its actual change is much slower than its sampling rate, the temporal locality assumption is correct. In the case of RS-fMRI with task-free, the voxel time series are usually modeled as convolution of neural activity and slowly changing HRF. From this view, the locality assumption can be justified (Yaesoubi, Adalı & Calhoun, 2017).

The proposed high-order FC network framework is based on sliding-window-based LoD-FCN, the parameters of the sliding window (i.e., *W*, *s*) inevitably affect the results, and the results shown in Fig. 4 also illustrate this point. For the RS-fMRI data used in this paper, each subject underwent a 6-minute scan and 180 volumes were acquired, i.e., per volume is sampled every 2 seconds. When the high-order FC network achieves good results, the value of *W* and *s* are relatively small, such as ***Ho*** (6) (*W* = 30, *s* = 2), ***Ho*** (8) (*W* = 30, *s* = 2) and ***Ho*** (10) (*W* = 30, *s* = 2) (see Table 2). It can be understood as that when the value of sliding window and translational step is too large, the number of temporal windows will be reduced, and the span of each moving is too large, which assumed that the mental activity of human brain will not change in a long time at resting state. And when the sliding window length and the translational step are large enough, the sliding-window-based LoD-FCN may degenerate into C-FCN.

### Compare with LoD-FCN

**Figure 6:**
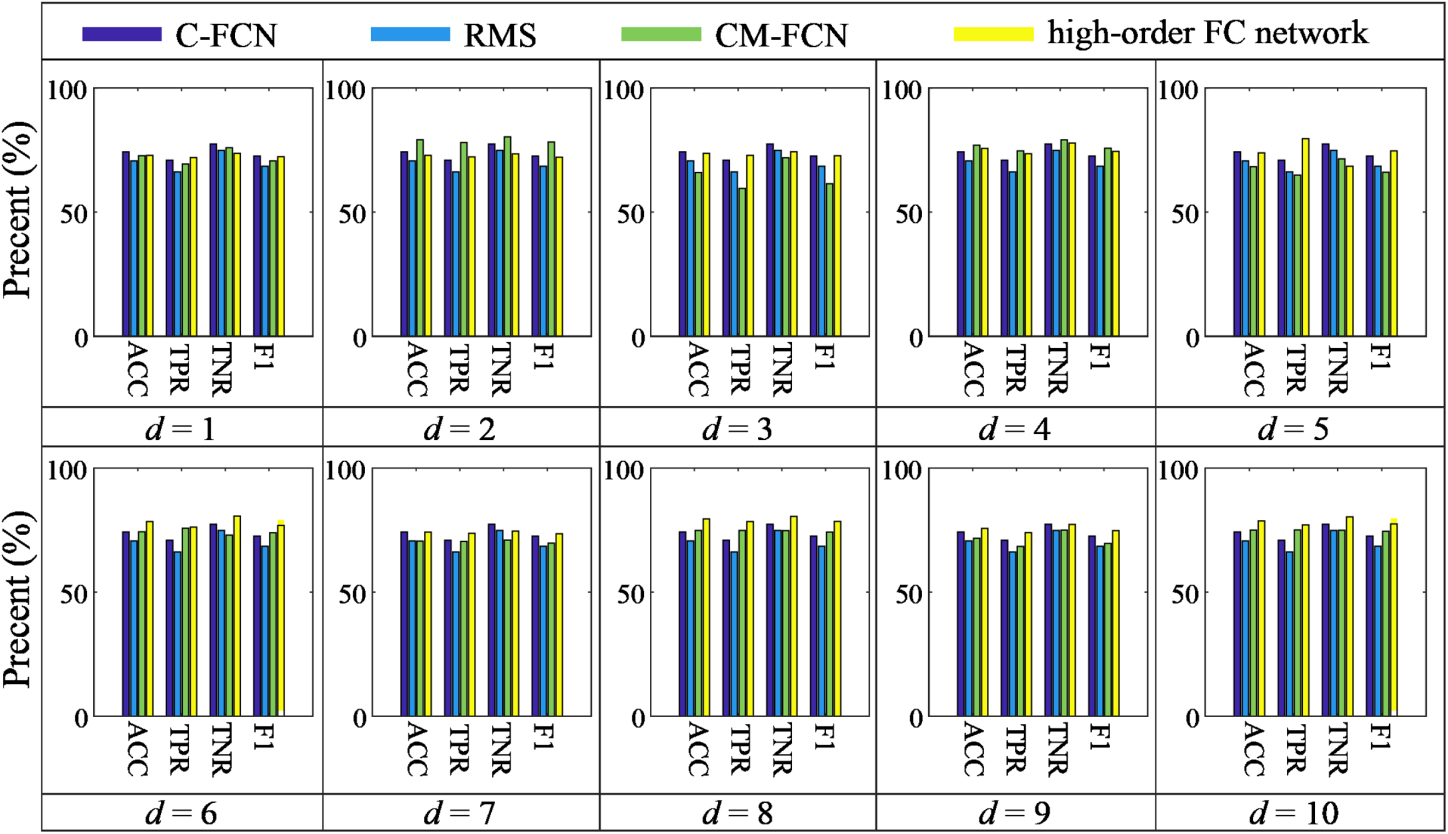
The histogram of our method compared with C-FCN, RMS and CM-FCNs.

In order to validate the effectiveness of our high-order FC network, we compare it with C-FCN and LoD-FCN. Specifically, the comparison method utilizes the C-FCN matrix and extracts statistical features based on central moment (i.e., CM-FCN) (Zhao et al., 2020) and root-mean-square (RMS) (Chen et al., 2017) from LoD-FCN as original features, and then perform feature selection for SVM classification. The best classification performances of comparison methods are summarized in Table 4. In order to compare with other methods intuitively, Fig. 6 shows the histogram of our method compared with other methods.

**Table 4:** The best performances of C-FCN, RMS and CM-FCN.

Features	*W*	*s*	ACC (%)	TPR (%)	TNR (%)	F1 (%)
*C-FCN*	–	–	74.29	70.89	77.40	72.58
*RMS*	70	16	70.63	66.22	74.89	68.48
*CM* (1)	40	16	72.72	69.33	75.93	70.63
*CM* (2)	60	10	79.18	78.00	80.33	78.24
*CM* (3)	90	16	65.95	59.56	71.93	61.44
*CM* (4)	60	10	76.92	74.67	79.11	75.74
*CM* (5)	30	14	68.27	64.89	71.47	66.07
*CM* (6)	70	10	74.36	75.78	73.00	73.93
*CM* (7)	40	12	70.60	70.44	70.96	69.76
*CM* (8)	70	10	74.86	74.89	74.84	74.18
*CM* (9)	40	16	71.75	68.44	75.04	69.66
*CM* (10)	70	10	75.01	75.11	74.98	74.51

As shown in Table 4 and Fig. 6, we can see that the classification performance of C-FCN is better than RMS and some CM-FCNs, such as ***CM*** (1), ***CM*** (3), etc., while the performance of ***CM*** (2) is better than C-FCN, RMS and other order CM-FCNs. It indicates that the feature type of LoD-FCN has a great influence on its classification performance. In addition, the classification performance of CM-FCN does not necessarily affect its corresponding high-order FC network. For instance, the ACC of ***CM*** (2) is 79.18%, while the ACC of higher-order FC network is 72.88%; the ACC of ***CM*** (8) is 74.86%, while the ACC of higher-order FC network is 79.50%. This indicates that CM-FCNs and high-order FC networks may provide complementary information in ASD diagnosis.

### Combination of FC networks with voting strategy

For the binary classification task, the learner }{}{E_t} will predict a label from the label set {}{}{c_1}, }{}{c_2}}, and the voting is the most common combination strategy. In this study, we adopt majority voting for combination, i.e., if a label gets more than half of the votes, it is predicted to be that label, otherwise, the prediction is rejected. Formally,

(9) }{}{f_j} = \left\{ {\matrix{ {{c_j},\; \; \; \; \; \; if\; \mathop \sum \nolimits_{t = 1}^T {E_t}\left( s \right) = {c_j} > \displaystyle{T \over 2};} \cr {reject,\; \; \; otherwise.} \cr } } \right.

where }{}{f_j} \in \left\{ {{c_1},{c_2}} \right\} denotes the label after the vote, }{}{E_t}\left( s \right) = {c_j} is the learner }{}{E_t} that predicts the label of the *s*-th subject as }{}{c_j}\; \left( {1 \le j \le 2} \right), *T* is the total number of the learners.

**Figure 7:**
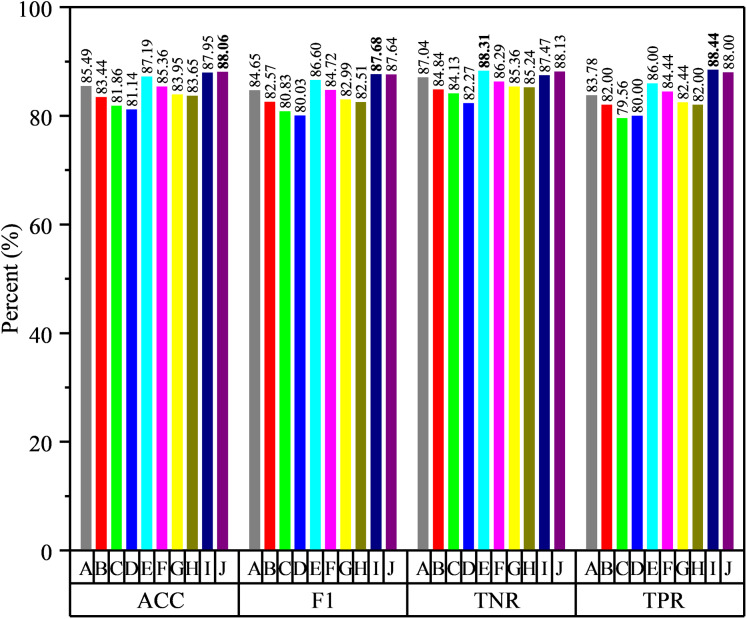
The results of voting strategies, where the specific strategies are shown in Table 5.

In general experience, in order to achieve good integration, individual learners should have certain accuracy and differences among learners. From Tables 2, 4 and Fig. 6, we can see that the classification accuracy of many classifiers is more than 70%, and there are obvious performance differences between them. Therefore, we use multiple networks to make voting decisions. The voting strategies are summarized in Table 5. Among them, ***C*** + ***CM*** (2) + ***Ho*** (8) denotes the combination of C-FCN, second-order CM-FCN and high-order FC network based on 8th order CM-FCN, ***RMS*** + ***CM*** (2) + ***Ho*** (8) denotes the combination of RMS feature extricated from LoD-FCN, 2nd-order CM-FCN and high-order FC network based on 8th order CM-FCN, the meanings of the other symbols are similarly defined. The results of voting combinations are summarized in Fig. 7.

**Table 5:** The voting with different feature type combinations.

Voting strategies	The abbreviations in Fig. 7
*C + CM* (2) *+ Ho* (8)	A
*RMS + CM* (2) *+ Ho* (8)	B
*CM* (2) + *CM* (4) + *CM* (10)	C
*Ho* (6) + *Ho* (8) + *Ho* (10)	D
*C* + *CM* (2) + *CM* (4) + *CM* (10) + *Ho* (8)	E
*RMS* + *CM* (2) + *CM* (4) + *CM* (10) + *Ho* (8)	F
*C* + *CM* (2) + *Ho* (6) + *Ho* (8) + *Ho* (10)	G
*RMS* + *CM* (2) + *Ho* (6) + *Ho* (8) + *Ho* (10)	H
*C* + *CM* (2) + *CM* (4) + *CM* (10) + *Ho* (6) + *Ho* (8) + *Ho* (10)	I
*RMS* + *CM* (2) + *CM* (4) + *CM* (10) + *Ho* (6) + *Ho* (8) + *Ho* (10)	J

From Fig. 7, we can see that the classification performance is improved by voting strategy fusion, while the fusion of different features improves the performance in different degrees. It indicates that the available functional correlation information from single FC network is limited and the FC information from different feature types can provide complementary information in ASD diagnosis. For example, the classification accuracy of RMS feature extracted from LoD-FCN is only 70.63%, which is lower than that of C-FCN. However, after merging with second-order CM-FCN and high-order FC network based on 8th order CM-FCM, the classification accuracy is higher than any one of them. Moreover, we note that the integration of different features from the same network improve indistinctly performance. For instance, the highest classification accuracy using a single CM-FCN is 79.18%, but the accuracy of ***CM*** (2) + ***CM*** (4) + ***CM*** (10) is 81.86%, only 2.28% higher than 2nd order CM-FCN, and the accuracy of ***Ho*** (6) + ***Ho*** (8) + ***Ho*** (10) only 1.64% higher than 8th high-order FC network.

In summary, through the voting strategy, we can draw the following conclusions: (1) by combining, the performance has been significantly improved and the highest accuracy has reached 88.06%; (2) the features from the same network may be less complementary, and the fusion of different network types is more conducive to improve the accuracy; (3) the selection of classifiers plays a key role in the fusion result, which relies on the complementarity among classifiers.

### Analysis of discriminative brain regions

**Figure 8:**
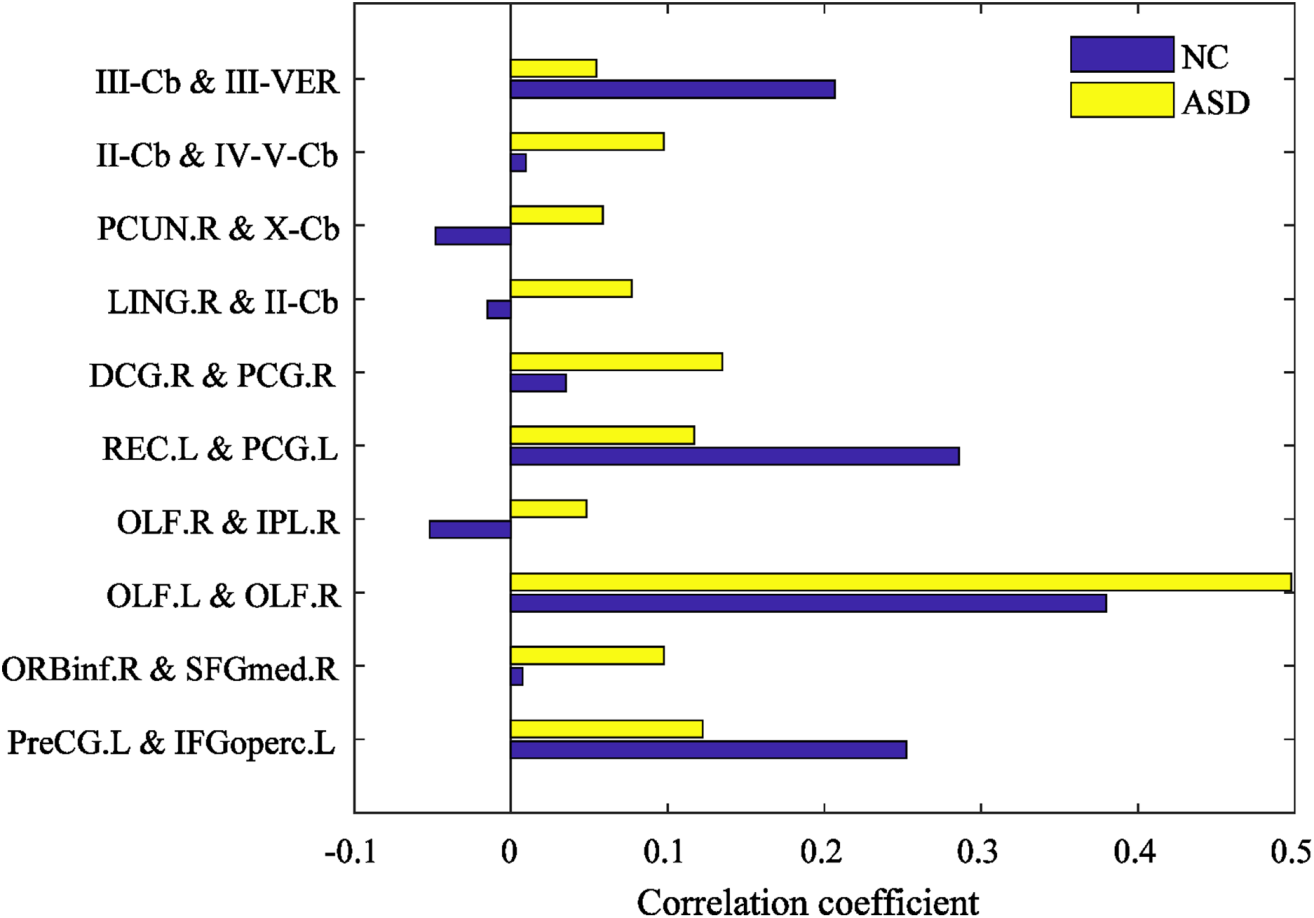
The mean high-order FC value of NC and ASD.

The mean correlation coefficients (high-order FCs) of the discriminative features are computed from NC and ASD children, respectively, which are shown in Fig. 8. There are great differences between NC and ASD high-order FC, both positive and negative correlations reflect genuine physiological processes (Goelman, Gordon & Bonne, 2014), the positive correlations reflect synchronized activity between brain regions, while negative correlations reflect a kind of anti-correlation or competitive relationship between brain regions (Fox et al., 2009; Uddin et al., 2010). Among the selected brain region pairs, the connections: PCUN.R & X-Cb, LING.R & II-Cb and OLF.R & IPL.R are showed positive and negative correlation on ASD and NC, respectively, indicating that the FCs of these connections may change from the original competitive relationship to the synchronous relationship. In previous studies, PCUN is one of the brain regions which predominate in DMN (Florian et al., 2013), and it is related to ASD (Urbain, Pang & Taylor, 2015); Cerebellum involving in the fine motor function (Hampson & Blatt, 2015), and it may also play an important role in cognition and emotion (Sui & Zhang, 2012); The OLF, which may serve in ASD intervention (Woo & Leon, 2013), may also provide for a novel early non-verbal non-task-dependent ASD marker (Rozenkrantz et al., 2015); LING is one of the brain regions responsible for visual processing; IPL is found to be linked to praxis development (Wymbs et al., 2021). The above studies suggest that these brain regions are associated with ASD. In the current study, the brain pairs: PCUN.R & X-Cb, LING.R & II-Cb and OLF.R & IPL.R are negative correlation in ASD, but positive in NC, indicating that these brain regions pairs changed from synchronous activity to competitive activity, which is a serious brain FC lesion.

Other discriminative brain region pairs have different degrees of connectivity strength changes, most of which come from DMN and CER. It is generally accepted that the DMN plays an important role in high-level cognitive functions, while abnormality of the DMN can be observed across a range of neurological disorders (Murdaugh et al., 2012; Washington et al., 2014). In the present study, the DMN is found that the failure of modulating the deactivation of the DMN and the abnormal connectivity of DMN with other regions have been found in ASD (Assaf et al., 2010; Kennedy, Redcay & Courchesne, 2006). PCUN.R, PCG.L, PreCG.L, OLF.R, SFGmed.R, REC.L belong to DMN. Besides the DMN, many brain regions of cerebellum are selected, such as II-Cb, III-VER and so on. Some recent studies have implicated cerebellar connectivity deficits in ASD patients (Khan et al., 2015; Igelström, Webb & Graziano, 2017; Wang, Kloth & Badura, 2014). These selected brain regions are consistently shown to be related to ASD pathology in the previous studies (Urbain et al., 2016; Ha et al., 2015).

### Analysis of connectivity between functional networks

Some intra-network and inter-network connections are abnormal in ASD, and these abnormal connections are important for understanding ASD diagnosis. Some studies have found that abnormal intra-network and inter-network connectivities in ASD (Liu & Huang, 2020; Morgan et al., 2019; von dem Hagen et al., 2013). In the current study, the discriminative brain regions are distributed over several common resting-state networks, and the connections distributed over intra-network and inter-network, such as PCUN.R and OLF.R belong to DMN, X-Cb belong to CER, IPL.R belong to EAN (see Fig. 5).

**Figure 9:**
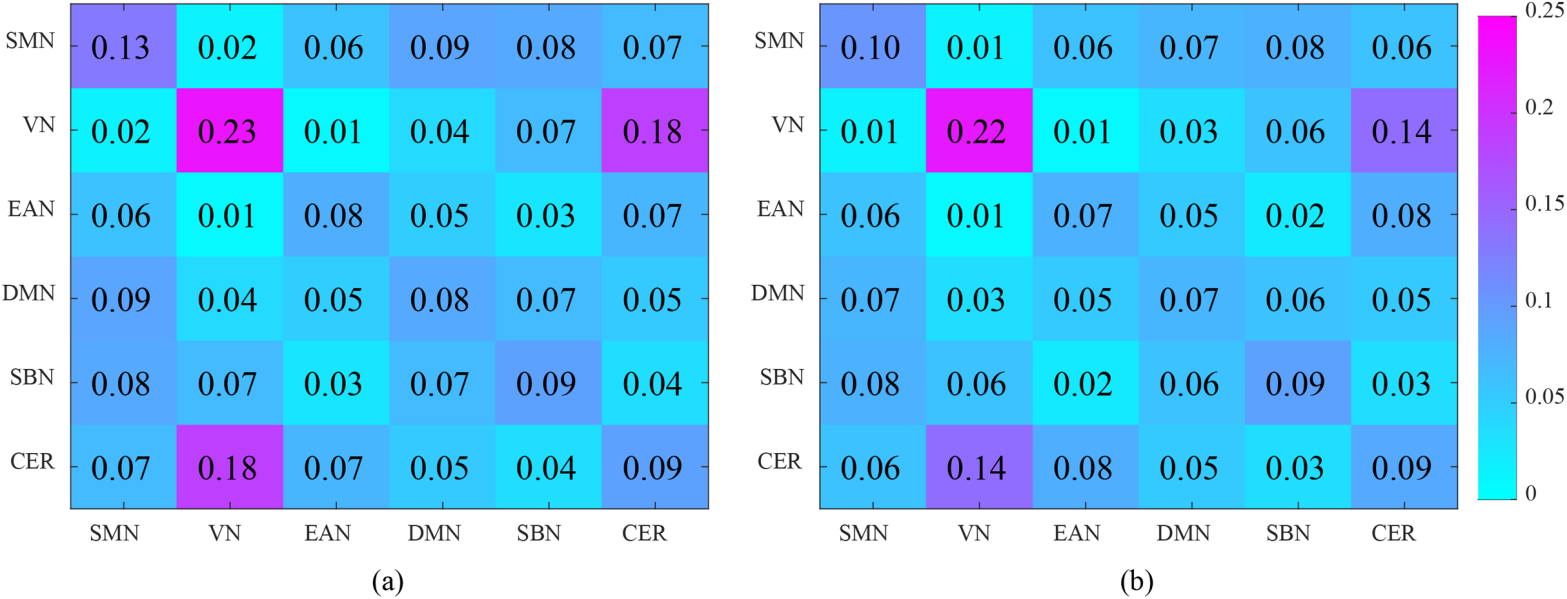
Analysis of the interaction between the six functional networks. (A) and (B) show the interaction matrix between different brain networks of NC and ASD, respectively.

Figure 9 shows the interactions between the six functional networks. Figs. 9A and 9B show the interaction matrix between two networks of NC and ASD, respectively, that is, the average of the FCs between all of the brain regions in one network and all of the brain regions in the other networks. As depicted in Fig. 9, the average connectivity strength inside SMN of NC is higher than ASD, and the interaction between VN and CER of NC is significantly higher than that of ASD. This shows that the connectivity strength between some functional networks of ASD is reduced, which is reflected in the intra-network and inter-network.

The SMN is a large-scale brain network that is activated during motor tasks and plays an important role in ASD-related studies. The social communication barriers, atypical sensory responsivity, repetitive and restrictive behaviors and other behavioral defects related to SMN have been included in the present ASD diagnostic criteria (Hannant et al., 2016). Recent studies have shown that ASD patients show abnormal development of the motor system (Hannant, Tavassoli & Cassidy, 2016; Mosconi & Sweeney, 2015; Tavassoli, Hoekstra & Baron-Cohen, 2014; Floris et al., 2016). For example, Mosconi & Sweeney (2015) suggested that sensorimotor deficits occur before social and communication deficits and are primary features of ASD. Tavassoli, Hoekstra & Baron-Cohen (2014) indicated that reduced sensory perception is associated with a greater number of autism symptoms. In the current study, the result also shows that connectivity within SMN deficits in ASD patients, which manifest as connectivity strength decreased.

The VN is responsible for the visual processing of human brain, and some brain regions of VN have been found abnormal in ASD, such as right fusiform gyrus (Urbain et al., 2016) and left calcarine (Perkins et al., 2015). The cerebellum plays an important role in both higher cognitive functions and motor control and coordination (Hampson & Blatt, 2015). Some studies have shown that have cerebellar connectivity defects in ASD patients (Igelström, Webb & Graziano, 2016; Khan et al., 2015). In our study, the average connectivity strength within VN and CER in ASD patients was slightly lower than that in NC patients. However, the interaction between VN and CER manifests as significant connectivity strength decreased. This indicates that the intra-network interaction between VN and CER networks of ASD patients is abnormal.

### Limitations

Our study has some limitations. Firstly, we employed the voting strategy based on classifier-level fusion to fuse the network, which limits our exploration of which brain regions have an impact on the fusion results. The discriminative brain regions play an important role in the detection of functional connectivity abnormalities in ASD. In the future work, we will explore feature-level fusion strategy to find useful discriminative brain regions for ASD classification. Secondly, the sample size limits our study. ABIDE database involved 17 international sites, among which the site NYU with the most data has 184 subjects (79 ASDs and 105 NCs). After our data preprocessing, only 92 subjects (45 ASDs and 47 NCs) were retained. Such a small sample size seriously limits our research on the generalization performance of the model, which is a common problem in the current research on ASD classification.

## Conclusion

In this paper, we proposed a novel high-order FC network framework, which is based on the central moment features of the low-order dynamic FC network. The developed method is simple and effective. It can not only avoid the time sensitive problem of dynamic network, but also capture the high-order connectivity patterns among brain regions. The experiments on ASD identification show that the high-order FC networks based on different order central moments have certain complementarity, and the classification accuracy can be improved by effective combination. The proposed high-order FC network is combined with other networks by voting strategies, and the classification accuracy reaches 88.06%. We found that some connectivity deficits in ASD patients, especially the directional changes of high-order FC in PCUN.R & X-Cb, LING.R & II-Cb and OLF.R & IPL.R brain region pairs, and the connectivity within SMN, and the interaction between VN and CER networks deficits in ASD patients.

## Supplemental Information

10.7717/peerj.11692/supp-1 Supplemental Information 1 Data Format.

10.7717/peerj.11692/supp-2 Supplemental Information 2 The RS-fMRI data.

10.7717/peerj.11692/supp-3 Supplemental Information 3 MATLAB code.

## Additional Information and Declarations

### Data Availability

The following information was supplied regarding data availability: The raw data file is available as a Supplemental File.
