# Supplementary material for: Constructing high-order functional connectivity network based on central moment features for diagnosis of autism spectrum disorder
Source: PeerJ. 2021 Jul 6;9:e11692. doi: 10.7717/peerj.11692 (PMC8269664; doi:10.7717/peerj.11692)
Supplement: Supplemental Information 1 [file peerj-09-11692-s001.docx]

In this study, we conducted experiments on the Autism Brain Imaging Data Exchange (ABIDE) database. ABIDE is a grassroots consortium aggregating and openly sharing 1112 existing resting-state functional magnetic resonance imaging (RS-fMRI) data sets with corresponding structural MRI and phenotypic information from 539 individuals with ASDs and 573 age-matched normal controls (NCs, 7–64 years). In this study, we chose 45 ASD patients (36 males and 9 females) and 47 NC subjects (36 males and 11 females) aged between 7 and 15 years old, scanned at New York University (NYU) Langone Medical Center. In the **2.1** section (i.e., **Data Acquisition and Preprocessing**) of the submitted manuscript, we introduced the process of data processing.

The data used in this paper are packaged in the “row data.zip” file. The “row data” file contains the RS-fMRI data of 92 subjects, i.e., Subject_1.csv to Subject_92.csv, and the “labels” file contains the corresponding tags of 92 subjects, where 1 represents ASD subjects and -1 represents NC subjects.
